# Fast Green FCF Attenuates Lipopolysaccharide-Induced Depressive-Like Behavior and Downregulates TLR4/Myd88/NF-κB Signal Pathway in the Mouse Hippocampus

**DOI:** 10.3389/fphar.2019.00501

**Published:** 2019-05-08

**Authors:** Jing Yang, Rongjun Liu, Fan Lu, Fang Xu, Jinwei Zheng, Zhao Li, Wei Cui, Chuang Wang, Junfang Zhang, Shujun Xu, Wenhua Zhou, Qinwen Wang, Junping Chen, Xiaowei Chen

**Affiliations:** ^1^Zhejiang Key Laboratory of Pathophysiology, School of Medicine, Ningbo University, Ningbo, China; ^2^Department of Anesthesiology, Ningbo No. 2 Hospital, Ningbo, China; ^3^Department of Anesthesiology, Shanghai East Hospital, Tongji University School of Medicine, Shanghai, China

**Keywords:** Fast green FCF, depression, TLR4, NF-κB, lipopolysaccharide

## Abstract

Depression is a common neuropsychiatric disorder and new anti-depressive treatments are still in urgent demand. Fast Green FCF, a safe biocompatible color additive, has been suggested to mitigate chronic pain. However, Fast green FCF’s effect on depression is unknown. We aimed to investigate Fast green FCF’s effect on lipopolysaccharide (LPS)-induced depressive-like behavior and the underlying mechanisms. Pretreatment of Fast green FCF (100 mg/kg, i.p. daily for 7 days) alleviated depressive-like behavior in LPS-treated mice. Fast green FCF suppressed the LPS-induced microglial and astrocyte activation in the hippocampus. Fast green FCF decreased the mRNA and protein levels of Toll-like receptor 4 (TLR4) and Myeloid differentiation primary response 88 (Myd88) and suppressed the phosphorylation of nuclear factor-κB (NF-κB) in the hippocampus of LPS-treated mice. Fast green FCF also downregulated hippocampal tumor necrosis factor (TNF)-α, interleukin (IL)-1β, and IL-6, but did not alter the level of the brain-derived neurotrophic factor (BDNF) in the hippocampus of LPS-treated mice. The molecular docking simulation predicts that Fast green FCF may interact with TLR4 and interrupt the formation of the TLR4-MD2 complex. In conclusion, the anti-depressive action of Fast green FCF in LPS-treated mice may involve the suppression of neuroinflammation and the downregulation of TLR4/Myd88/NF-κB signal pathway in mouse hippocampus. Our findings indicate the potential of Fast green FCF for controlling depressive symptoms.

## Introduction

Depression is one of the most common neuropsychiatric disorders and becomes a world-wide serious health problem. Multiple lines of evidence from human studies indicate a positive correlation between neuroinflammation and depression (Najjar et al., 2013; Benatti et al., 2016; Miller and Raison, 2016). Increased microglial activation and production of inflammatory mediators, such as tumor necrosis factor (TNF)-α, interleukin (IL)-1β, and IL-6, have been found in post-mortem brain samples from suicide depressed patients (Pandey, 2017). Epidemiological studies also report high rates of comorbidity between depression and inflammatory diseases, indicating that inflammation induces depressive symptoms in at-risk individuals (Kiecolt-Glaser et al., 2015; Benatti et al., 2016; Marrie et al., 2017). Consistently, plenty of preclinical studies also show that administration of lipopolysaccharide (LPS) or pro-inflammatory cytokines cause a phase of depressive-like behavior, which is strongly correlated with the robust neuroinflammation in the brain (Rossol et al., 2011; Cazareth et al., 2014). These investigations suggest the potential of anti-inflammatory therapies in the treatment of depression.

Toll-like receptor 4 (TLR4) is an important member of the TLR family, which is intensively expressed in the immune system and plays vital roles in immune responses (Lu et al., 2008). Activation of TLR4 by LPS induces phosphorylation of the nuclear factor-κB (NF-κB) through the Myeloid differentiation primary response 88 (Myd88) adaptor, and enhances expression of inflammatory mediators, e.g., IL-1β, IL-6, IL-15, and IL-27 in the brain (Poltorak et al., 1998; Lu et al., 2008; Gorina et al., 2011). Other studies provide additional evidence that the TLR4/NF-κB pathway is activated in the frontal cortex and the hippocampus in stress-induced depression in mice (Garate et al., 2013; Wang et al., 2018). Consistently, stress-induced depressive behavior, NF-κB activation, upregulation of the pro-inflammatory mediators, and cell damage in frontal cortex and hippocampus are reduced in TLR4-deficient mice (Garate et al., 2013; Cheng et al., 2016). These outcomes suggest an essential role of TLR4 in the pathogenesis of depression.

Fast Green FCF is approved by the U.S. Food and Drug Administration as a color additive used to color food, drugs, and cosmetics. Fast green FCF exhibits a high degree of safety since its acceptable daily intake is up to 25 mg/kg in human (Borzelleca and Hallagan, 1992). Our previous study demonstrates that Fast green FCF could mitigate complete Freund’s adjuvant (CFA)-induced pain. The anti-nociceptive effect of Fast green FCF may involve downregulation of spinal purinergic P2X4 receptor (P2X4R), a key player in the development of chronic pain (Xu et al., 2018). However, it leaves questions open as to whether and how Fast green FCF affects inflammation-induced depressive symptoms.

In the present study, we aimed to examine the anti-depressive effect of Fast green FCF on LPS-treated mice because systemic LPS treatment is commonly used to study inflammation-induced depressive-like behaviors in rodents. We then investigated whether the Fast green FCF’s action involves P2X4R, TLR4/Myd88/NF-κB, pro-inflammatory cytokines (TNF-α, IL-1β, and IL-6), and the brain-derived neurotrophic factor (BDNF) in the hippocampus. Our work contributes to the discovery of potential biocompatible treatments of neuroinflammation and the inflammation-associated depression.

## Materials and Methods

### Animals

Male ICR mice (8 – 10 weeks, 20 – 25 g) purchased from Experimental Animal Center of Zhejiang Province (Hangzhou, China) were used in this study. The animals were housed in a temperature-controlled animal facility with a 12 h light–dark cycle. All procedures were approved by the Animal Care and Use Committee of Ningbo University in accordance with the guideline for the Care and Use of Laboratory Animals by National Institutes of Health (NIH Publications No. 80-23).

### Experimental Design

**FIGURE 1:**
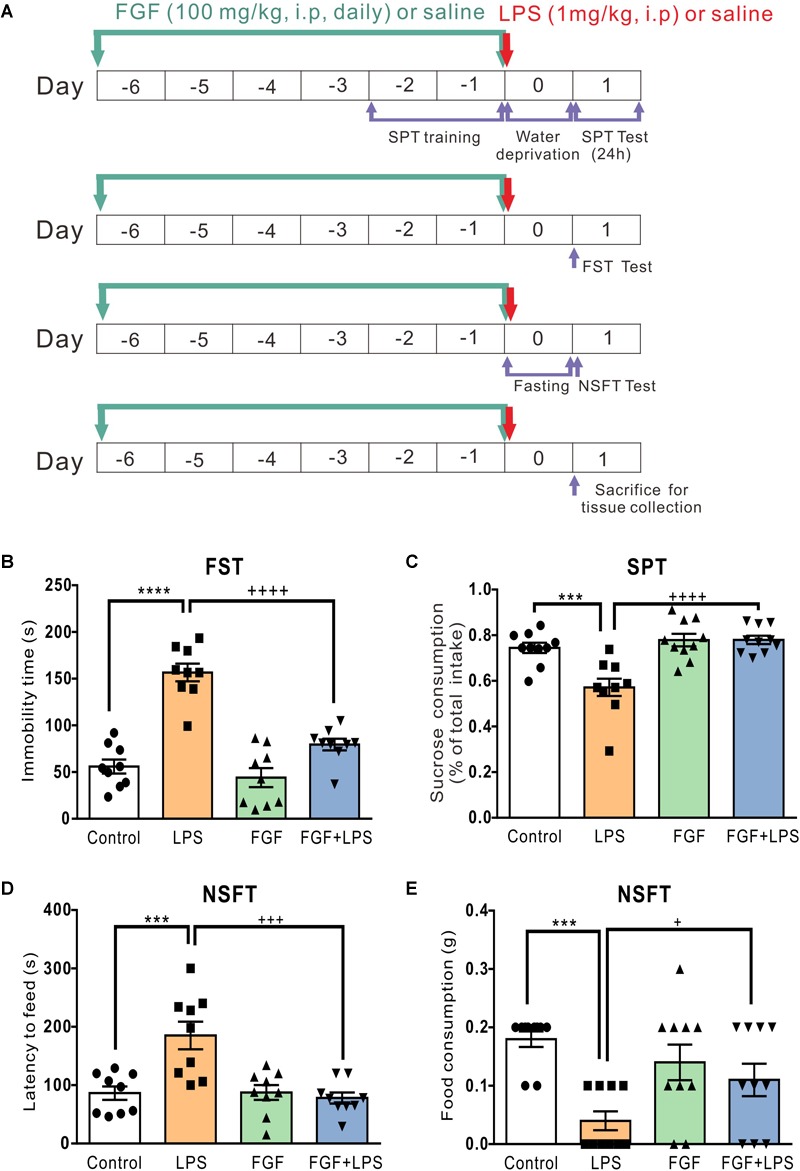
Fast green FCF reduced LPS-induced depressive-like behavior in mice. **(A)** The diagram of the experimental timeline. Mice received Fast green FCF (100 mg/kg, i.p.) or vehicle (saline) injections daily for 7 days (from the day –6 to day 0). LPS (1 mg/kg, i.p.) was applied immediately after the last Fast green FCF injection on day 0. Forced swimming test (FST), Sucrose preference test (SPT), and Novelty-suppressed feeding test (NSFT) were conducted independently to avoid interference. On day 1 (24 h post-LPS), mice were sacrificed for biological assays. **(B)** Fast green FCF decreased the immobility time in LPS mice at 24 h post-LPS. **(C)** Fast green FCF increased the sucrose preference in LPS mice (between 24 h and 48 h post-LPS). **(D)** Fast green FCF decreased the latency to feed in LPS mice at 24 h post-LPS. **(E)** Fast green FCF increased the amount of food consumption in LPS mice at 24 h post-LPS. Fast green FCF, FGF. Data are presented as means ± SE. Two–way ANOVA followed by Turkey’s *post hoc* test. ^∗∗∗^, ^∗∗∗∗^ vs controls and +, +++, ++++ vs LPS-treated animals; one symbol *p* < 0.05, three symbols *p* < 0.001, and four symbols *p* < 0.0001 (*n* = 9–10 mice/group).

The experimental timeline of drug administration and behavioral tests is shown in Figure 1A. In order to prevent interference between behavioral experiments, experiments were performed independently. Freshly dissolved Fast green FCF in saline (0.9% NaCl) or the vehicle (saline) was injected intraperitoneally daily for 7 days before LPS treatment (day -6 to 0). On day 0, LPS (1 mg/kg, i.p.) or the vehicle (saline) was injected. Fast green FCF and LPS were purchased from Sigma-Aldrich (St. Louis, MO, United States).

### Depression Behavioral Tests

#### Forced Swimming Test (FST)

The test was performed as previously described. Briefly, 24 h after LPS injection, the FST was carried out in a soundproof room. Mice were individually placed into the center of a glass cylinder (15 cm diameter, 30 cm height). The water depth was around 20 cm to prevent mice from touching the cylinder bottom with tails or limbs. The water temperature was maintained at 23–25°C. Black cardboards were placed between every two cylinders to mitigate the interaction of mice. Each mouse was allowed to swim for 6 min. All test sessions were videotaped by a digital camera. The immobility time during the last 4 min was scored offline. The immobility time was defined as only when they were motionless or passively floating on the water (with only movements necessary for keeping balance).

#### Sucrose Preference Test (SPT)

Mice were singly housed and habituated with one bottle of sucrose (1%, w/v) and one bottle of water for 48 h before LPS treatment (from the day-2 to day-1). Bottle positions were switched after 24 h. After LPS treatment, mice were then water deprived for 24 h immediately. Sucrose preference test was conducted between 24 h and 48 h after LPS injection. During the test session, mice were singly housed and exposed to one bottle of 1% sucrose and one bottle of water for 24 h. Bottle positions were switched after 12 h. Total consumption of each fluid was measured and the sucrose preference was calculated according to the following ratio: sucrose preference (%) = [sucrose intake (g) / sucrose intake (g) + water intake (g)] × 100%.

#### Novelty-Suppressed Feeding Test (NSFT)

On day 0 after LPS injection, animals were deprived of all food in the home cage (fasting for 24 h). Water remained available *ad libitum*. After fasting, mice were placed in a holding cage and allowed to habituate for 30 min. A small piece of chow was placed in the center of a clear testing box (45 × 45 × 20 cm). The test began immediately after the animal was placed in a corner of the box. The feeding latency to begin feeding was recorded (the cut-off time was 5 min). Immediately after the mouse began to eat, the mouse was transferred back to its home cage. The amount of food consumed in 5 min was measured as food consumption.

### Enzyme-Linked Immunosorbent Assays (ELISA) Assay

Twenty-four hours after LPS injection, all animals were anesthetized by CO2 and then decapitated, the bilateral hippocampi were harvested, rinsed with cold saline and wiped by filter paper. Enzyme-linked immunosorbent assays (ELISA) were used to quantify BDNF (Cat. No.: DBNT00; R&D, Minneapolitan, United States), IL-1β (Cat. No.: MTA00B; R&D, Minneapolitan, United States), TNF-α (Cat. No.: EM001; ExCell Bio, Taicang, China), and IL-6 (Cat. No.: EM004; ExCell Bio, Taicang, China) concentrations. The preparation of all reagents, the working standards, and the protocol was followed according to the manufacturer’s instructions. The absorbance was read at 450 nm using a microplate spectrophotometer (Thermo Inc., United States).

### Real-Time Polymerase Chain Reaction (RT-PCR)

The RT-PCR analysis was performed as previously described (Xu et al., 2018). Briefly, mice were anesthetized with CO2 and sacrificed 24 h after LPS treatment. The mRNA of hippocampus tissue was extracted by the Trizol reagent (Invitrogen, Carlsbad, CA, United States) and then reverse-transcribed to complementary DNA using reverse transcriptase (Invitrogen, Carlsbad, CA, United States). The cDNA was then amplification using HiFiScript cDNA Synthesis Kit (CWBIO, Beijing, China) by ABI Q5 RT-PCR System (Applied Biosystems, Thermo Fisher, United States). Primers for TLR4 was: 5′- ATGGCATGGCTTACACCACC -3′ (forward), 5′- GAGGCCAATTTTGTCTCCACA -3′ (reverse); for Myd88 was: 5′- AGGACAAACGCCGGAACTTTT -3′ (forward), 5′- GCCGATAGTCTGTTCTAGT -3′ (reverse); for P2X4R was: 5′- GCTGCAGAAAACTTCACCCTC -3′ (forward), 5′- CATGATGCCTCCCTCCACTG -3′ (reverse); for NF-κB was: 5′- GGAGGCATGTTCGGTAGTGG -3′ (forward), 5′- CCCTGCGTTGGATTTCGTG -3′ (reverse); for GAPDH (the housekeeper gene): 5′- CATGGCCTTCCGTGTTCCTA -3′ (forward), 5′- TACTTGGCAGGTTTCTCCAGG -3′ (reverse). All primers were synthesized by BGI Co., Ltd (Shenzhen, China).

### Western Blot

Twenty-four hours after LPS injection, the mice were sacrificed. The hippocampi were collected and homogenized in lysis buffer containing protease inhibitors (Cat. No.: A32961.SJ2434041; Thermo Scientific, Carlsbad, United States). Samples were separated on 10% SDS-PAGE gels and transferred to PVDF membrane (0.22 μm; Millipore, Temecula, CA, United States). The membrane was blocked with 5% non-fat dry milk in Tris-buffered saline and then incubated overnight with rabbit anti-P2X4R (1:100, Cat. No.: APR002AN1802; Alomone Labs, Israel), TLR4 (1:1000, Cat. No.: 14358.1; CST, Massachusetts, United States), MyD88(1:1000, Cat. No.: 4283.4; CST, Massachusetts, United States), NF-κB (1:1000, Cat. No.: 8242.9; CST, Massachusetts, United States), or p-NF-κB (1:1000, Cat. No.: 3303.16; CST, Massachusetts, United States). The mouse anti-β-actin monoclonal (1:1000, Cat. No.: 4ab000001.4AH271811F; 4A Biotech, Beijing, China) antibodies were used as internal control. After washes, the membrane was then incubated with 800 CW goat anti-rabbit IgG antibody (1:15000, Cat. No.: 92632211.C20906-02; LI-COR, Nebraska, United States) and 800 CW goat anti-mouse IgG antibody (1:15000, Cat. No.: 92632210.C20808-02; LI-COR, Nebraska, United States) for 60 minutes. Target bands were revealed with a fluorescence scanner (Odyssey Infrared Imaging System, LI-COR Biotechnology, NE, United States). Western blots were analyzed using Image J analysis software (NIH, United States) to quantify the bands.

### Immunohistochemistry

Twenty-four hours after LPS injection, the mice were anesthetized with 4% chloral hydrate and then perfused transcardially with saline followed by 4% formaldehyde at room temperature. The brains were dissected out, post-fixed in 4% formaldehyde overnight and then transferred to sucrose (20% for 24 h and then 30% for 24 h). Brains were frozen in OCT compound (Sakura Finetek, CA, United States) and cut into 25-μm-thick sections (CM1950, Leica, Frankfurt, Germany). The sections containing hippocampus region were incubated at 4°C overnight in 0.1 M PBS buffer containing 0.5% Triton X-100 and the primary antibodies: mouse anti-GFAP (1:300, Cat. No.: 3670.6; CST, Massachusetts, United States), goat anti-Iba-1(1:500, Cat. No.: ab5076.GR3195324-1; Abcam, Cambridge, United States). Sections were then washed 3 times (8–10 min) in PBS solution and incubated for 90 min at room temperature in the same buffer containing Alexa 488-conjugated secondary antibodies: goat anti-mouse (1:200, Cat. No.: CW0113S.30251; CWBIO, Beijing, China), donkey anti-goat (1:500, Cat. No.: ab150129.GR3198891-1; Abcam, Cambridge, United States). The slides were mounted and viewed with a confocal laser scanning microscope (SP8, Leica, Frankfurt, Germany). Quantification was performed by ImageJ software (NIH, United States). In brief, the image background was subtracted and the threshold was set by ImageJ. Related hippocampal regions were then manually outlined as regions of interest (ROIs), and the total fluorescent intensities in the ROIs per unit area were calculated. The relative fluorescent intensity was the fold change compared to the control group. For each group, images were obtained from at least three mice.

### Molecular Docking

The molecular docking analysis was performed as previously described (Xu et al., 2018). Briefly, the analysis was performed by the SYBYL software (Tripos Inc., St. Louis, MO, United States) to predict the possible interaction between Fast green FCF and TLR4-MD2 complex. The crystal structure of mouse TLR4-MD2 complex was obtained from the protein data bank (PDB code: 2Z64) based on the study of Kim et al. (2007). The Fast green FCF’s structure was created by standard geometric parameters of SYBYL, and then optimized by the Powell method. The Surflex–Dock program was employed to perform docking analysis (Jain, 2003).

### Data and Statistical Analysis

Data are presented as means ± SE. Analyses were performed using the software package GraphPad Prism 5 (GraphPad Software, San Diego, CA, United States). Two–way analysis of variance (ANOVA) followed by Turkey’s *post hoc* tests as used for analyzing statistic difference as indicated in Figure legends. *p* < 0.05 was considered as statistically significant.

## Results

### Systemic Pre-administration of Fast Green FCF Alleviated the Depressive-Like Behavior in LPS-Inflamed Mice

The experimental timelines of drug administration and behavioral tests are shown in Figure 1A. Consistent with previous studies using the same animal model, LPS-treated mice exhibited the increased immobility time in FST (Figure 1B), the decreased sucrose preference in SPT (Figure 1C), the increased feed latency (Figure 1D), and the decreased food consumption (Figure 1E) in NSFT.

According to our previous study, we found that the anti-nociceptive effect of Fast green FCF was accumulative and a single Fast green FCF application did not reduce inflammatory pain. In addition, systemic administration of 100 mg/kg Fast green FCF had a more rapid and significant effect than 30 mg/kg Fast green FCF on inflammatory pain, and the anti-nociceptive effect of 100 mg/kg Fast green FCF persisted after the discontinuation of daily treatment (Xu et al., 2018). Therefore, we here investigated the anti-depressive effect of 100 mg/kg Fast green FCF administration (daily for 7 days, before LPS injection) on LPS-treated mice because LPS-induced depression-like behaviors occurred at 24 h after LPS treatment. As shown in Figure 1, Fast green FCF reduced the immobility time (Figure 1B), increased the sucrose preference (Figure 1C), reduced the feed latency (Figure 1D), and increased the food consumption (Figure 1E) in LPS mice.

Interestingly, we found that Fast green FCF did not affect LPS-induced sickness behavior. For instance, Fast green FCF did not affect the increase of immobility time in the tail suspension test (2 h post-LPS, Supplementary Figures S1A,B), the reduction of rearing and line crossing behaviors in open field test (6 h post-LPS, Supplementary Figures S1C–F), or the drop of core body (anal) temperature (0 – 6 h post-LPS; Supplementary Figure S2). These outcomes suggest that Fast green FCF did not prevent the induction of the LPS-induced sickness responses (2–6 h post-LPS), but rather facilitated the recovery from sickness and alleviates the following depressive behaviors, such as the learned helplessness and anhedonia (24 h post-LPS).

### Fast Green FCF Suppressed LPS-Induced Activation of Microglia and Astrocytes in the Dentate Gyrus (DG), Cornu Ammonis (CA) 1 and CA3 Regions of Mouse Hippocampus

It has been intensively reported that inflammatory responses in the central nervous system are initiated by activated glial cells (Dheen et al., 2007). To investigate the effect of Fast green FCF on activation of microglia and astrocytes, the main glial cells in the CNS, we examined the expression of activated microglial marker ionized calcium binding adaptor molecule-1 (Iba-1) and astrocyte marker glial fibrillary acidic protein (GFAP) in mouse hippocampus.

**FIGURE 2:**
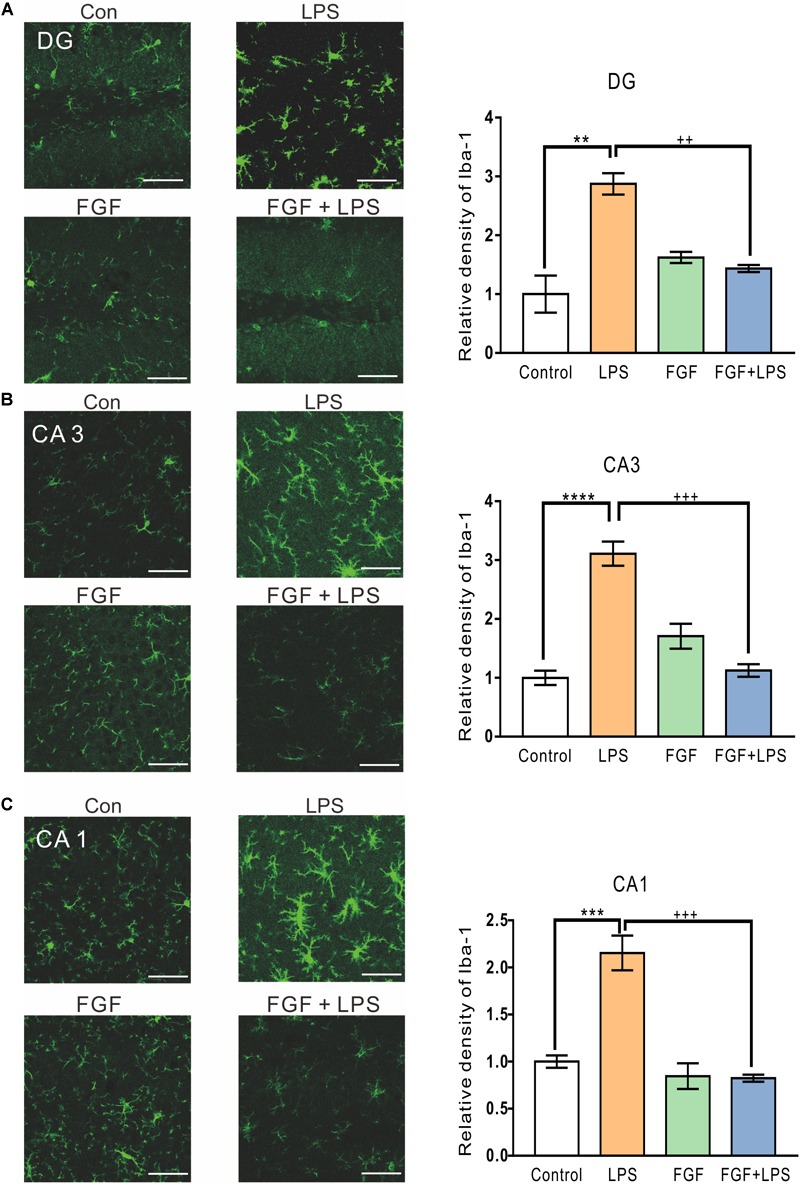
Fast green FCF suppressed LPS-induced microglial activation in DG, CA3, and CA1 regions of the mouse hippocampus. Representative images (left) and the statistic result (right) of Iba-1 staining in the hippocampal DG **(A)**, CA3 **(B)**, and CA1 **(C)** regions. Scale bar = 50 μm. Fast green FCF, FGF. Data are expressed as fold change (normalized to control) and presented as means ± SE. Two–way ANOVA followed by Turkey’s *post hoc* test. ^∗∗^, ^∗∗∗^, ^∗∗∗∗^ vs controls and ++, +++ vs LPS-treated animals; two symbols *p* < 0.01, three symbols *p* < 0.001, and four symbols *p* < 0.0001 (*n* = 3 mice/group).

The levels of Iba-1 in hippocampal DG (Figure 2A), CA3 (Figure 2B), and CA1 (Figure 2C) regions were elevated in the LPS-inflamed mice. Pre-administration of Fast green FCF decreased Iba-1 levels in these regions (Figure 2).

**FIGURE 3:**
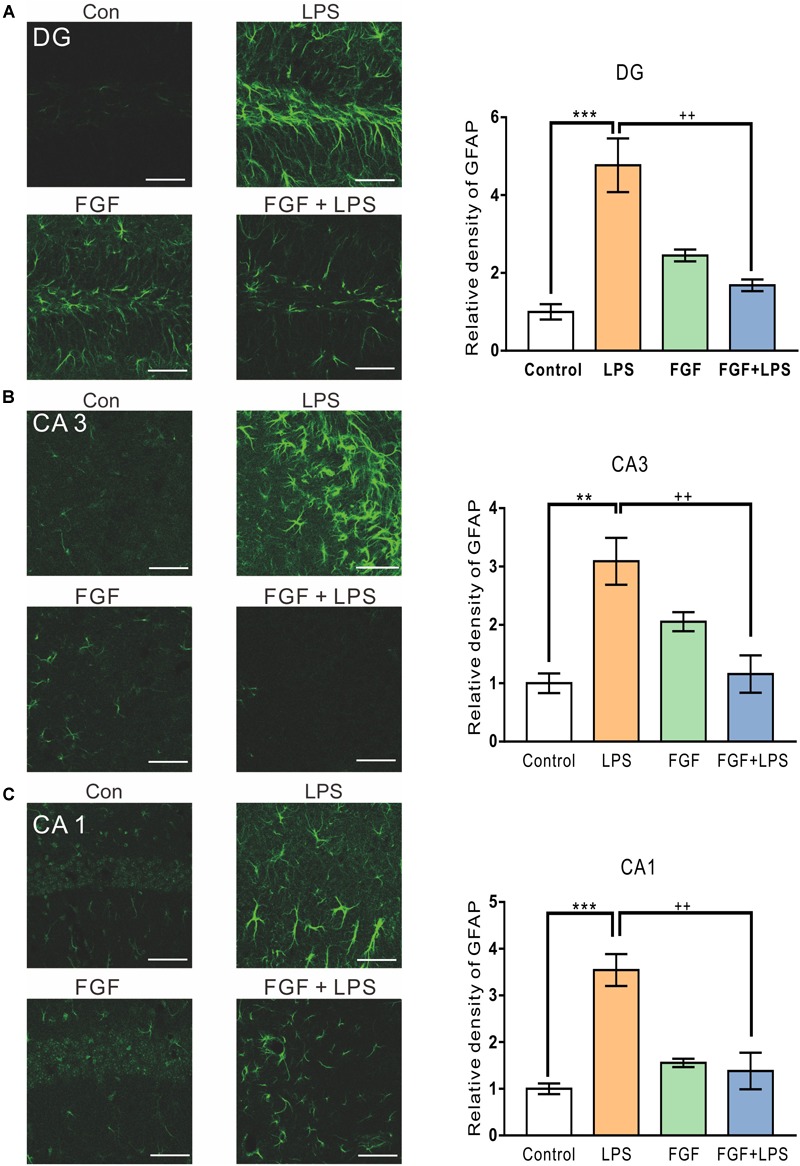
Fast green FCF suppressed LPS-induced astrocyte activation in DG, CA3, and CA1 regions of the mouse hippocampus. Representative images (left) and the statistic result (right) of GFAP staining in the hippocampal DG **(A)**, CA3 **(B)**, and CA1 **(C)** regions. Scale bar = 50 μm. Fast green FCF, FGF. Data are expressed as fold change (normalized to control) and presented as means ± SE. Two–way ANOVA followed by Turkey’s *post hoc* test. ^∗∗^, ^∗∗∗^ vs controls and ++ vs LPS-treated animals. Two symbols *p* < 0.01, three symbols *p* < 0.001 (*n* = 3 mice/group).

Similarly, the LPS-elevated expression of GFAP was also decreased by Fast green FCF in hippocampal DG (Figure 3A), CA3 (Figure 3B), and CA1 (Figure 3C) regions. Thus, our findings suggest an inhibitory effect of Fast green FCF on LPS-induced glial activation in mouse hippocampus.

### Fast Green FCF Down-Regulated the Expression of TLR4 and Myd88, as Well as the Phosphorylation of NF-κB in the Hippocampi of LPS-Inflamed Mice

**FIGURE 4:**
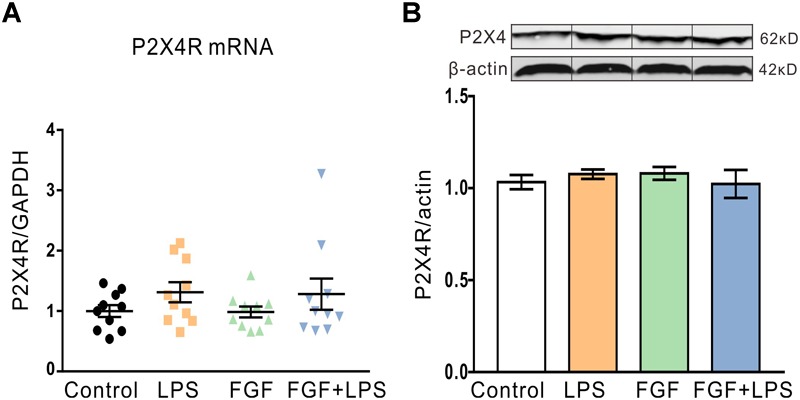
The P2X4R mRNA and protein levels in the hippocampus were not altered after LPS and/or Fast green FCF treatments. **(A)** The level of hippocampal P2X4R mRNA transcription after LPS and/or Fast green FCF treatment. GAPDH was served as internal standard. **(B)** The level of hippocampal P2X4R protein expression after LPS and/or Fast green FCF treatment. β-actin was served as internal standard. Fast green FCF, FGF. Data are expressed as fold change (normalized to control) and presented as means ± SE. Two–way ANOVA followed by Turkey’s *post hoc* test (mRNA: *n* = 10 mice/group; protein: *n* = 3 mice/group).

Our previous study suggests the involvement of spinal P2X4R in the anti-nociceptive action of Fast green FCF in a mouse model of CFA-induced inflammatory pain. Previous studies also approve abundant expression of P2X4R in rodent hippocampal neurons and glial cells (Stokes et al., 2017). Here, we investigated the effect of Fast green FCF on hippocampal P2X4R mRNA and protein levels in LPS-treated mice. Unexpectedly, neither the P2X4R mRNA transcription (Figure 4A) nor the protein translation (Figure 4B) changed at 24 h post-LPS injection, suggesting that LPS or Fast green FCF did not alter the P2X4R expression in the mouse hippocampus.

**FIGURE 5:**
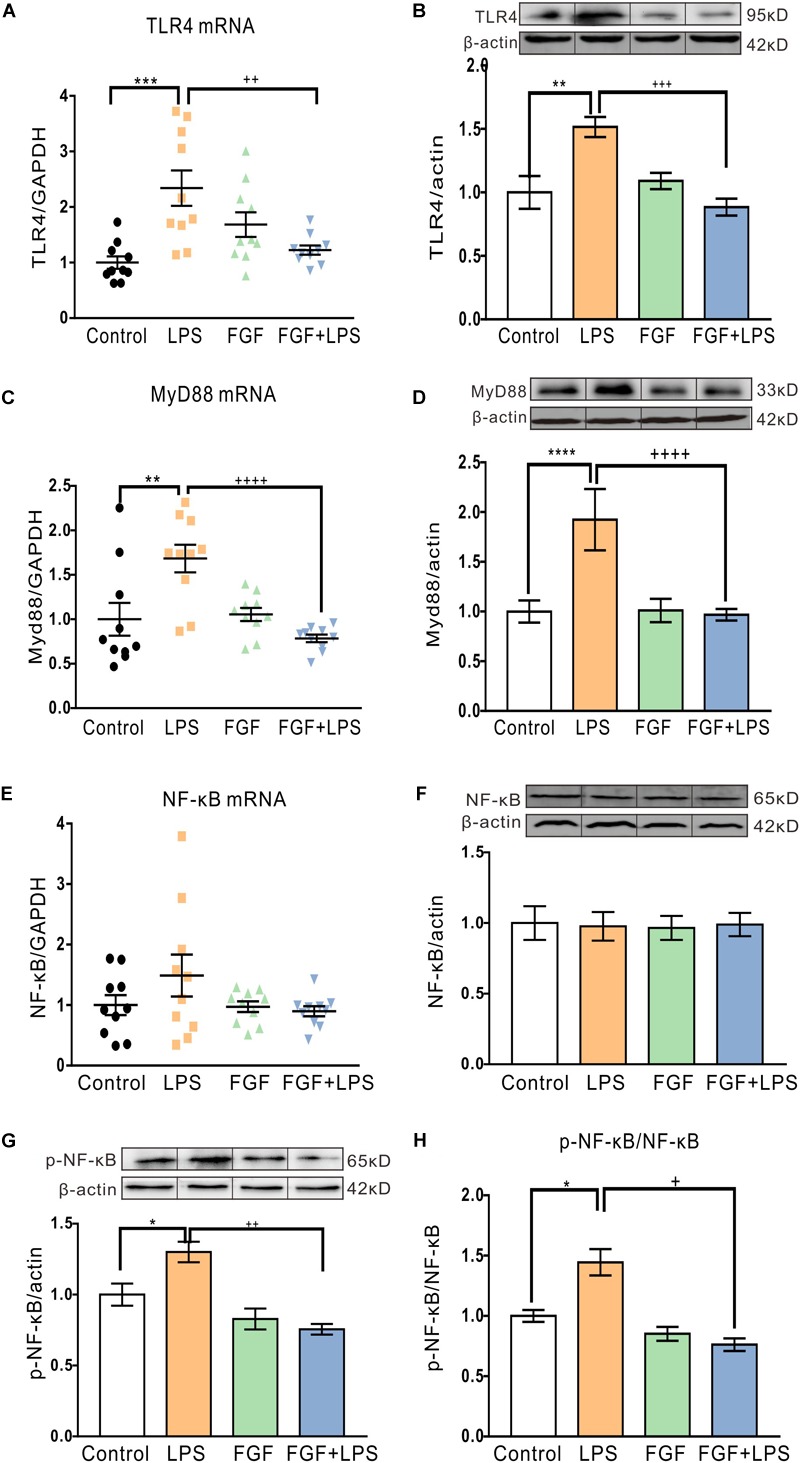
Fast green FCF decreased LPS-elevated TLR4 and Myd88 mRNA and protein levels and suppressed phosphorylation of NF-κB in the mouse hippocampus. **(A,B)** Fast green FCF decreased LPS-enhanced hippocampus TLR4 mRNA **(A)** and protein **(B)** levels. **(C,D)** Fast green FCF decreased LPS-enhanced hippocampus Myd88 mRNA **(C)** and protein **(D)** levels. **(E,F)** The levels of hippocampal NF-κB mRNA **(E)** and protein **(F)** after LPS and/or Fast green FCF treatment. **(G)** The level of hippocampal phosphorylated NF-κB (p65) protein after LPS and/or Fast green FCF treatment. **(H)** The ratio of phosphorylated NF-κB to total NF-κB. GAPDH or β-actin was served as an internal standard of mRNA or protein, respectively. Fast green FCF, FGF. Data are expressed as fold change (normalized to control) and presented as means ± SE. Two–way ANOVA followed by Turkey’s *post hoc* test. ^∗^, ^∗∗^, ^∗∗∗^, ^∗∗∗∗^ vs controls and +, ++, +++, ++++ vs LPS-treated animals. one symbol *p* < 0.05, two symbols *p* < 0.01, three symbols *p* < 0.001, four symbols *p* < 0.0001 (mRNA: *n* = 10 mice/group; protein: *n* = 3 mice/group).

Previous studies have verified that TLR4 is directly activated by LPS and initiates Myd88-dependent downstream cascades to activate glial cell activation in the central nervous system (Gorina et al., 2011; Shao et al., 2018). Therefore, we tested the Fast green FCF’s action in regulating hippocampal TLR4/Myd88 transcription and translation post-LPS treatment. LPS-evoked inflammation enhanced hippocampal TLR4 mRNA and protein levels (Figure 5A,B). As expected, pre-administration of Fast green FCF decreased the elevated TLR4 mRNA (Figure 5A) and protein levels (Figure 5B), as well as Myd88 mRNA (Figure 5C), and protein levels (Figure 5D) in the hippocampi of LPS-treated mice.

Extensive evidence shows that phosphorylation of NF-κB is an essential downstream event following TLR4 activation. Although LPS did not alter the NF-κB mRNA transcription and expression (Figure 5E,F), it enhanced the phosphorylation of NF-κB (p65) in the hippocampi of LPS mice (Figure 5G). Fast green FCF treatment inhibited the enhanced phosphorylation of NF-κB in LPS mice (Figure 5G,H), suggesting the effect of Fast green FCF on the suppression of the NF-κB activation in mouse hippocampus post-LPS.

### Fast Green FCF Decreased the Enhanced Production of Pro-inflammatory Cytokines (IL-1β, IL-6, and TNF-α), but did Not Affect the BDNF Level in the Hippocampi of LPS-Inflamed Mice

**FIGURE 6:**
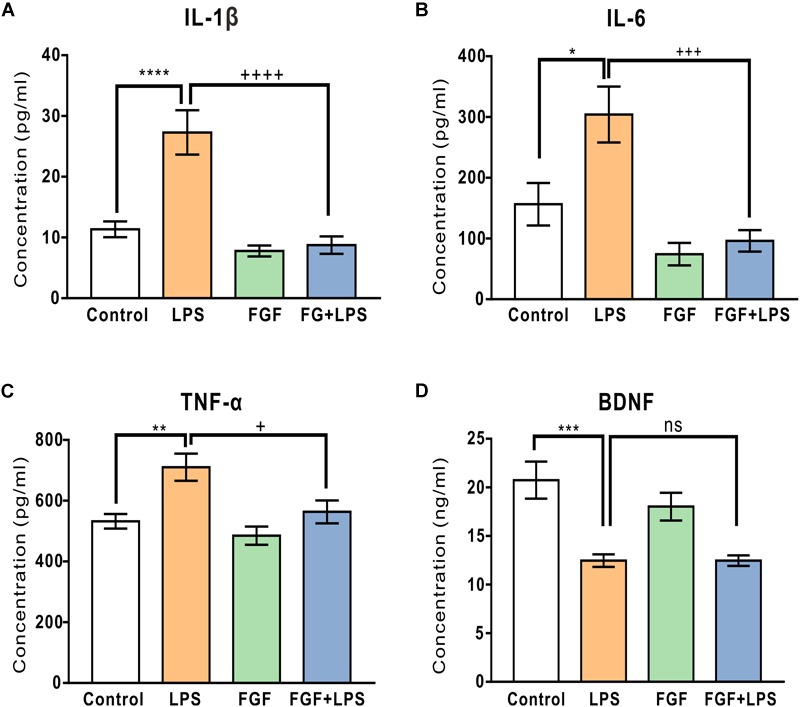
Fast green FCF reduced LPS-elevated IL-1β, IL-6, and TNF-α levels, but did not affect LPS-suppressed BDNF level in the mouse hippocampus. **(A–C)** Fast green FCF decreased LPS-enhanced hippocampal pro-inflammatory cytokine IL-1β **(A)**, IL-6 **(B)**, and TNF-α **(C)** levels. **(D)** Fast green FCF did not reverse the reduction of BDNF level post-LPS injection. Fast green FCF, FGF. Data are presented as means ± SE. Two–way ANOVA followed by Turkey’s *post hoc* test. ^∗^, ^∗∗^, ^∗∗∗^, ^∗∗∗∗^ vs LPS-treated animals. ++, +++, ++++ vs LPS-treated animals; one symbol *p* < 0.05, two symbols *p* < 0.01, three symbols *p* < 0.001, four symbols *p* < 0.0001 (*n* = 8–10 mice/group).

Levels of hippocampal cytokine IL-1β, IL-6, and TNF-α were determined at 24 h post-LPS injection. As expected, LPS injection caused a marked increase in hippocampal IL-1β (Figure 6A), IL-6 (Figure 6B), and TNF-α levels (Figure 6C). Fast green FCF reduced LPS-elevated IL-1β, IL-6, and TNF-α levels in the hippocampus (Figure 6A–C). Although LPS injection caused a significant decrease in hippocampal BDNF concentration, Fast green FCF did not enhance the hippocampal BDNF level in LPS mice (Figure 6D).

### The Molecular Docking Simulation Indicates an Interaction Between Fast Green FCF and TLR4

**FIGURE 7:**
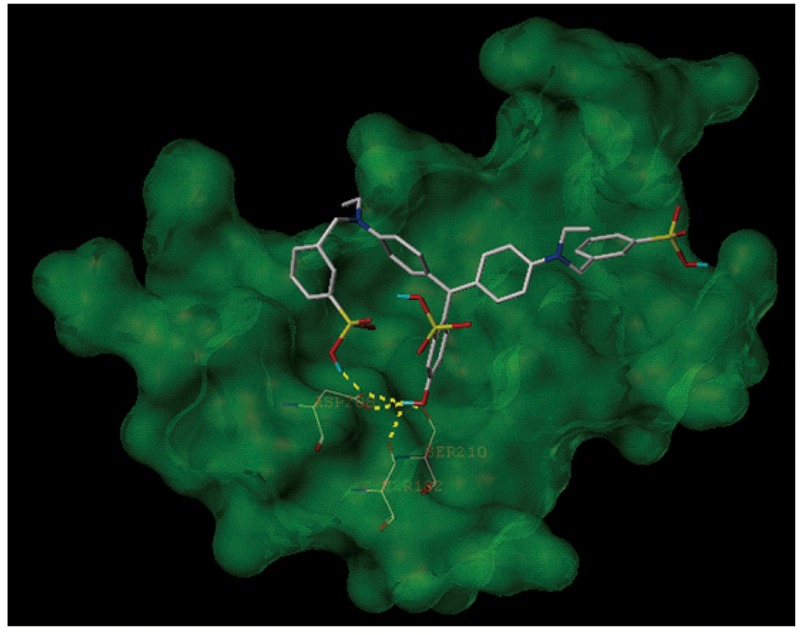
The molecular docking simulation of the interaction between Fast green FCF and the mouse TLR4-MD-2 complex. The low-energy conformation of Fast green FCF bound to TLR4-MD-2 complex (dark green). Fast green FCF is depicted as a stick model showing carbon (white), oxygen (red), nitrogen (dark blue), sulfate (yellow), and hydrogen (light blue). Yellow dash lines represent hydrogen bonds.

Because TLR4 and myeloid differentiation factor 2 (MD-2) form a heterodimer that recognizes LPS, Fast green FCF may interrupt LPS’s activity through inhibiting the formation of the TLR4-MD-2 complex. We thus applied a molecular docking simulation to predict any potential interaction between Fast green FCF and the mouse TLR4-MD-2 complex. Molecular docking analysis of the low-energy conformation of Fast green FCF predicts that Fast green FCF may form hydrogen bonds with side chains of Ser182, Asp208, and Ser210 of TLR4 (Figure 7). Because the sites of Asp208 and Ser182 are very close to critical binding sites of TLR4 with MD-2, the simulation suggests that Fast green FCF may obstruct the stable formation of TLR4-MD2 complex, and consequently influence TLR4 downstream signal cascade.

## Discussion

**FIGURE 8:**
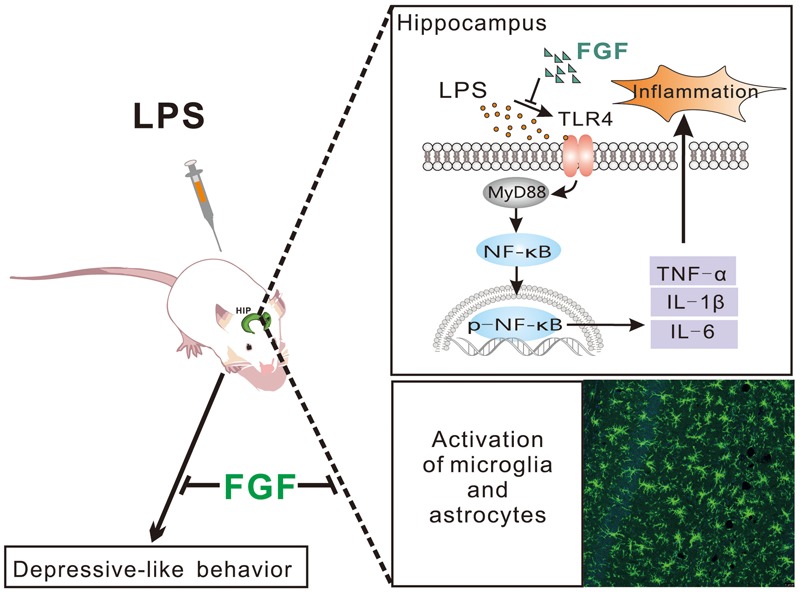
Schematic diagram of potential mechanisms of Fast green FCF’s anti-depressive action post-LPS exposure.

Preclinical evidence has demonstrated that LPS-induced depressive symptoms relate to the activation of astrocytes and microglia, and overproduction of pro-inflammatory cytokines, such as TNF-α, IL-1β, and IL-6 in the brain, especially in the rodent hippocampus *in vivo* and *in vitro* (Rossol et al., 2011; Cazareth et al., 2014). In the present study, we showed the effect of Fast green FCF against the LPS-induced depressive-like behaviors. The anti-depressive mechanisms may involve the reduction of microglial and astrocyte activation, the downregulation of TLR4 and Myd88 expression, the decreases of NF-κB phosphorylation and pro-inflammatory TNF-α, IL-1β, and IL-6 levels in the hippocampus (Figure 8).

Although the LPS treatment induces transient depressive-like behaviors, it causes the long-lasting elevation of pro-inflammatory cytokines and neuronal loss in the brain (>10 months) (Qin et al., 2007). It has been intensively reported that suppression of neuroinflammation in the brain (e.g., hippocampus) reduces depressive-like symptoms in different animal models of depression (Brites and Fernandes, 2015; Colasanti et al., 2016; Kim et al., 2016; Zhou et al., 2017). Clinical studies also confirmed the anti-depressive effect of anti-inflammatory agents and TNF inhibitor infliximab in depressed patients (Raison et al., 2013; Kohler et al., 2014). Therefore, control of systemic inflammation may help to reduce the risk of depression. Considering the effect of Fast green FCF on activation of glial cells and suppression of inflammatory mediators, such as pro-inflammatory cytokines and NF-κB, Fast green FCF may have potential to treat depression patients. Furthermore, since FGF has been used in food industries for many years and exhibits a high degree of biosafety, FGF has potential to improve depressive symptoms and may increase treatment response to conventional antidepressants.

A growing body of evidence shows that TLR4 pathway may be an important link between inflammation and depression. Increased expression of TLR4 has been found in post-mortem brain samples from depressed suicide victims (Pandey et al., 2014, 2018). 4 weeks of antidepressant treatment has been shown to significantly decrease blood TLR1-9 expression, as well as depressive symptoms in MDD patients (Hung et al., 2016). Moreover, a recent clinical study has shown that the mRNA transcription of TNFAIP3, a suppressor of the TLR4 pathway, was inversely correlated with severity of depression and effectively predicted response to antidepressant treatment in major depressive disorder (Hung et al., 2017). In addition, animal studies have also pointed out a vital role of TLR4 signal pathway in different animal models exhibiting depressive-like behavior. TLR4 knockout mice are less susceptible to depressive-like behavior induced by learned helplessness (Cheng et al., 2016). Activation of glycogen synthase kinase-3 (GSK3), NF-κB, and the NLRP3 inflammasome in the hippocampus might be associated with the depressive-like behavior in the learned helplessness model (Cheng et al., 2016). It has also been reported that upregulation of TLR4 signal pathway is involved in depression associated with other diseases, such as Alzheimer’s disease, obesity and alcohol addiction (Strekalova et al., 2015; Ledo et al., 2016; Anton et al., 2017; Wang et al., 2018). Ledo et al. (2016) have found that soluble Amyloid-beta oligomers, a key player in the pathogenesis of Alzheimer’s disease, fail to induce depressive-like behavior in TLR4-deficient mice and in mice harboring a non-functional TLR4 variant in myeloid cells. In addition, mice receiving a high-cholesterol diet (0.2%) exhibit depression- and anxiety-like behaviors and upregulation of TLR4 in the prefrontal cortex and the liver (Strekalova et al., 2015). Similarly, leptin-deficient ob/ob mice also display depressive-Like behaviors and an enhanced TLR4/NF-κB signal in the frontal cortex and hippocampus (Wang et al., 2018). Alcohol binge administration produces depressant-like effects during acute withdrawal and enhances TLR4/NF-κB signal cascades in the frontal cortex (Anton et al., 2017). These findings indicate the TLR4-mediated pathway might be a common pathway leading to depressive-like symptoms induced by immune disorders. In the present study, we have found that Fast green FCF significantly downregulates the LPS-enhanced TLR4/Myd88/NF-κB signal pathway in the hippocampus. Previous studies have shown that Ser183 and Asp209 of TLR4 could form a hydrogen bond with Arg106 of MD-2, and Asp209 is essential in the polar interaction of the formation of stable TLR4-MD-2 complex (Han et al., 2012; Rehman et al., 2018). Our docking simulation predicts that Fast green FCF may form hydrogen bonds with Ser182 and Asp208 of TLR4, which are close to the binding site of TLR4 (Ser183 and Asp209) with MD-2. The simulation outcome indicates that Fast green FCF may obstruct the stable formation of the TLR4-MD-2 complex, and thus inhibit the downstream Myd88/NF-κB cascades.

Our previous investigation suggests the relationship between Fast green FCF’s anti-nociceptive action and the downregulation of spinal P2X4R expression in CFA-induced inflammatory pain (Xu et al., 2018). Furthermore, some studies show that P2X4R is abundantly expressed in glial cells of the hippocampus and LPS facilitates P2X4R-mediated inward currents (Stokes et al., 2017). Unexpectedly, we here found that LPS did not increase hippocampal P2X4R transcription and expression. One possible explanation is that the hippocampal P2X4R level does not increase in response to LPS at 24 h after LPS treatment. However, we could not completely exclude the contribution of P2X4R to Fast green FCF’s action because Fast green FCF may regulate P2X4R channel activity at the functional level.

Various preclinical and clinical investigations suggest a correlation between downregulation of hippocampal BDNF and pathogenesis of depression (Bjorkholm and Monteggia, 2016; Zhang et al., 2016). Although we found that LPS decreased hippocampal BDNF concentration, Fast green FCF did not change the reduction of BDNF in LPS mice. The results are consistent with our previous finding that Fast green FCF did not alter the spinal BDNF level in CFA-treated mice (Xu et al., 2018). Our outcomes suggest that the anti-inflammatory and anti-depressive action of Fast green FCF may not relate to the BDNF pathway in the hippocampus.

## Conclusion

In conclusion, our results demonstrate that Fast green FCF has anti-inflammatory and anti-depressive effects against LPS treatment. The mechanism involves the inhibition of glial activation and cytokine releases through TLR4/Myd88/NF-κB signal pathway in the hippocampus. Our findings suggest the potential of Fast green FCF in helping to control depressive symptoms, and as an adjuvant therapy for the major depressive disorder.

## Ethics Statement

This study was carried out in accordance with the guideline for the Care and Use of Laboratory Animals by National Institutes of Health (NIH Publications No. 80-23). The protocol was approved by the Animal Care and Use Committee of Ningbo University.

## Author Contributions

JY contributed to animal experiments, analysis and interpretation of the data, and drafted the manuscript. RL, FL, and FX contributed to animal experiments, analysis and interpretation of the data, and writing of the manuscript. JiZ, ZL, CW, JuZ, WZ, and QW contributed to molecular experiments, analysis and interpretation of the data, and writing of the manuscript. WC and SX contributed to the molecular docking experiment, analysis and interpretation of the data, and writing of the manuscript. JC supervised the study and contributed to the conception and design of the study. XC supervised the study and contributed to the conception and design of the study, the analysis and interpretation of the data, and writing of the manuscript. All authors approved the final version of the manuscript.

## Conflict of Interest Statement

The authors declare that the research was conducted in the absence of any commercial or financial relationships that could be construed as a potential conflict of interest.
