# Supplementary material for: Fast Green FCF Attenuates Lipopolysaccharide-Induced Depressive-Like Behavior and Downregulates TLR4/Myd88/NF-κB Signal Pathway in the Mouse Hippocampus
Source: Front Pharmacol. 2019 May 8;10:501. doi: 10.3389/fphar.2019.00501 (PMC6519320; doi:10.3389/fphar.2019.00501)
Supplement: Supplementary file 1 [file Data_Sheet_1.pdf]

Supplementary Data

Fast green FCF attenuates lipopolysaccharide-induced depressive-like behavior and downregulates TLR4/Myd88/NF-κB signal pathway in the mouse hippocampus

Jing Yang[1], Rongjun Liu[1], Fan Lu[1], Fang Xu [2], Jinwei Zheng[2], Zhao Li[3], Wei Cui[1], Chuang Wang[1], Junfang Zhang[1], Shujun Xu[1], Wenhua Zhou[1], Qinwen Wang[1], Junping Chen[2]*, Xiaowei Chen[1]*

[1]Zhejiang Key Laboratory of Pathophysiology, School of Medicine, Ningbo University, Ningbo, China

[2] Department of Anesthesiology, Ningbo No. 2 Hospital, Ningbo, China

[3] Department of Anesthesiology, East Hospital, Tongji University School of Medicine, Shanghai, China

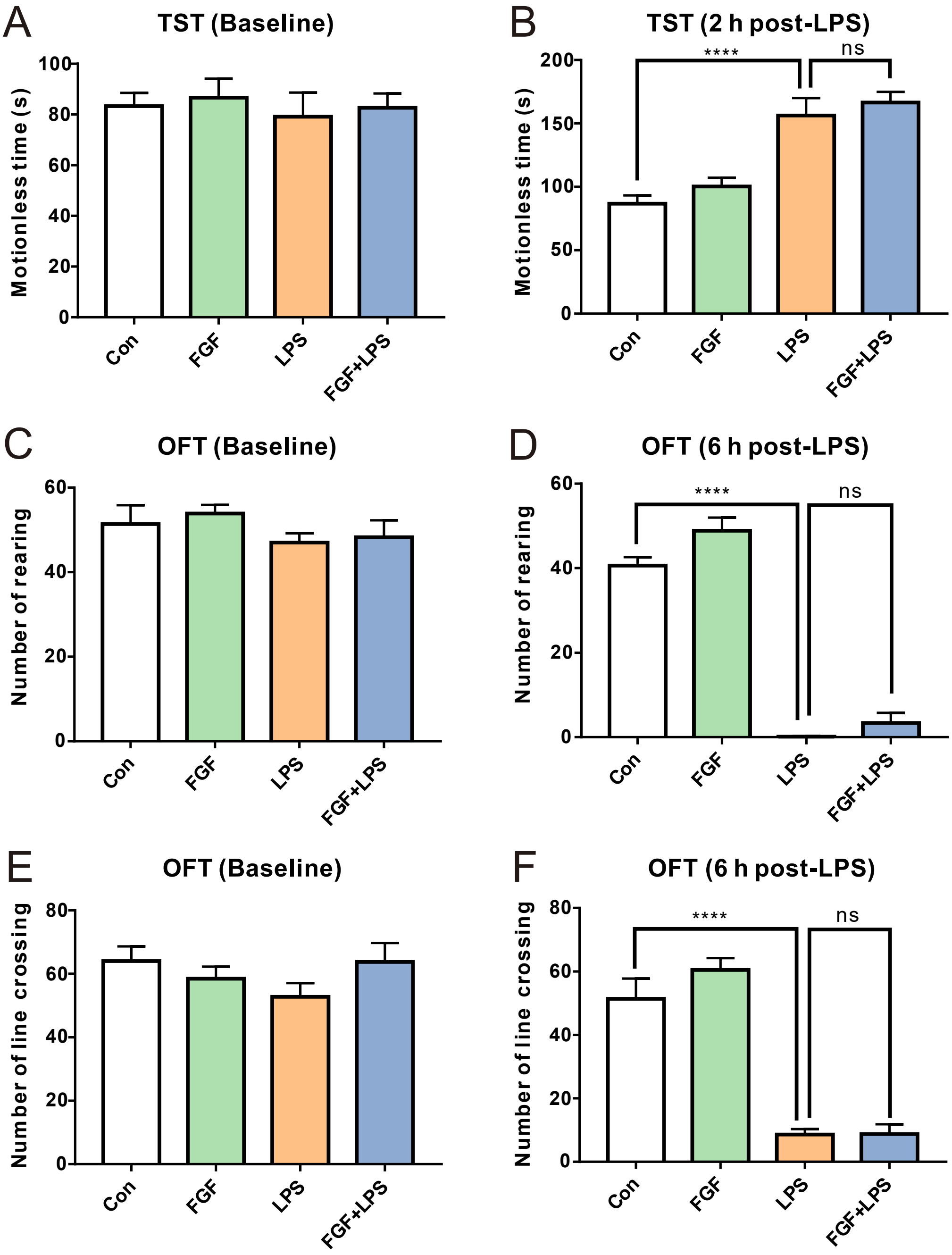

Figure S1: The effect of Fast green FCF (FGF) on LPS-induced sickness behavior. (A) The baseline of the motionless time measured by the tail suspension test (TST) before FGF treatment (Day -6). (B) FGF did not reduce the motionless time in LPS-treated mice at 2 h post-LPS. (C) The number of rearing measured during 5 min by the open filed test (OPT) before FGF treatment (Day -6). (D) FGF did not increase the number of rearing in LPS-treated mice at 6 h post-LPS. (E) The number of line crossing measured during 5 min by the open filed test (OPT) before FGF treatment (Day -6). (F) FGF did not increase the number of line crossing in LPS-treated mice at 6 h post-LPS. Data are presented as means ± SE. Two–way ANOVA followed by Turkey's post hoc test. **** vs controls, $p < 0.0001$ ($n = 8$ mice/group).

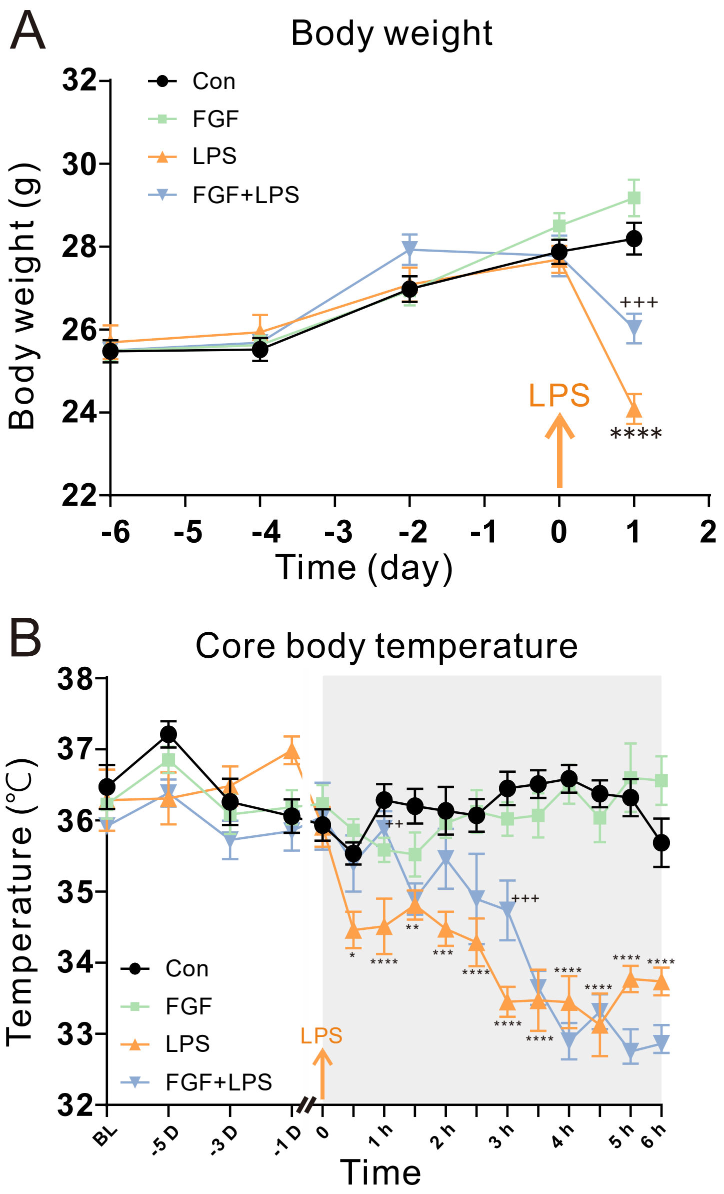

Figure S2: The effect of Fast green FCF (FGF) on the body weight (A) and the core body (anal) temperature (B) of control and LPS-treated mice.

(A) FGF prevented the loss of body weight of LPS-treated mice at day 1 (24 h post-LPS injection). (B) FGF did not increase the anal temperature of control and LPS-treated mice within 6 h post-LPS. BL, baseline. Data are presented as means ± SE. Two–way ANOVA followed by Turkey's post hoc test. *, **,***, **** vs controls and +++ vs LPS-treated animals; one symbol $p < 0.05$, two symbol $p < 0.01$, three symbols $p < 0.001$, four symbols $p < 0.0001$ ($n = 8$ mice/group).

Methods:

Tail suspension test (TST)

Mice were suspended 50 cm above the desktop with an adhesive tape placed at 1 cm from the tip of tails. The suspension was last for 6 min and the motionless time was recorded during the last 4 min.

Open field test (OFT)

The locomotor activity of the mice was assessed at 6 h post-LPS injection. The animals were individually placed in a white Plexiglas box (50 X 50 X 45 cm) in a dim room. The floor of the box was divided into four identical squares. Line crossings (with all four paws placed into a new square) and rearings (with both front paws raised from the floor) were recorded in a 5-min period.
